# Potential public health impact of the adjuvanted recombinant zoster vaccine among people aged 50 years and older in Beijing

**DOI:** 10.1080/21645515.2021.1932216

**Published:** 2021-07-26

**Authors:** Christa Lee, Ning Jiang, Haiwen Tang, Chiyu Ye, Yanfei Yuan, Desmond Curran

**Affiliations:** a GSK, Singapore, Singapore; b GSK, Beijing, China; c GSK, Wavre, Belgium

**Keywords:** herpes zoster, public health impact, vaccination, healthcare resource utilization, China, shingles, postherpetic neuralgia, older adults

## Abstract

Herpes zoster (HZ) is a painful, unilateral rash which occurs upon reactivation of latent varicella zoster virus due to age-related immunity decline or immuno-suppression. In 2019, the recombinant zoster vaccine (RZV) was the first vaccine to be approved in China for HZ prevention. This study aimed to estimate the potential public health impact of RZV vaccination, compared with the status quo of no vaccination, in individuals ≥50 years of age (YOA) in Beijing, by adapting the published ZOster ecoNomic Analysis (ZONA) model. We considered 5% and 50% vaccination coverage for the private market (near-term post-launch) and mass vaccination (long-term) settings respectively. In the base-case analysis of both market settings, second-dose compliance was set to 80%. Coverage and second-dose compliance rates were varied under scenario and sensitivity analyses. In the base case, mass vaccination with RZV was estimated to prevent 435,681 HZ cases, 51,558 postherpetic neuralgia (PHN) cases, and 15,703 cases of other HZ-related complications in the overall ≥50 YOA cohort over their remaining lifetime, compared with no vaccination. Under the same base-case scenario, 14,247 hospitalizations and 1,031,387 outpatient visits could be avoided. The 50–59 YOA cohort had the highest contributions to the overall reduction in HZ cases, its complications and related healthcare resource utilization. Results were robust under numerous scenario and sensitivity analyses. This analysis demonstrates the potential of RZV vaccination to substantially reduce the public health burden of HZ among individuals ≥50 YOA, and may inform appropriate vaccination strategies for HZ prevention, particularly in urban settings within China.

## Introduction

Herpes zoster (HZ) results from a reactivation of latent varicella-zoster virus (VZV) infection, which is believed to occur when VZV-specific cell-mediated immunity falls below a critical threshold due to aging or immuno-suppression.^1^ HZ is often characterized by a painful dermatomal vesicular rash; the majority of cases are uncomplicated, with symptoms resolving within 4–6 weeks.^2,3^ However, some patients experience complications, with the most common being postherpetic neuralgia (PHN), a persistent, often debilitating pain that can last several months to several years,^4,5^ which may affect 5–30% of individuals with HZ.^6^ Other complications, including ocular and neurological complications, also frequently occur during the acute phase of HZ and may result in long-term physical impairments.^4,7^ While mortality due to HZ is uncommon,^8^ HZ can greatly reduce patients’ quality of life by affecting physical, social, functional and psychological health, particularly amongst those with PHN.^4,9^ There may also be spill-over impacts on patients’ social circles, and in particular on family members and caregivers.^4,10^

The risk of HZ increases with advancing age, owing to age-related immunity decline against VZV.^11,12^ A systematic literature review focusing on China reported an HZ incidence ranging between 2.9–5.8 per 1,000 person-years among adults ≥50 years of age (YOA), suggesting a substantial burden of HZ in China comparable to the rest of the world.^6,13^ This is a particular concern in view of China’s aging population, where the proportion of adults ≥50 YOA is increasing by 3–4% annually.^13^ At least 2.8 million new cases may be expected annually across China, with a predicted 55,000 cases a year in Beijing city alone.^14^

Current treatment options remain limited; antivirals must be administered within 72 hours of symptom onset in order to be effective.^4^ Even if administered within 72 hours, they have limited efficacy in accelerating rash healing and relieving pain, and have not demonstrated efficacy in reducing the risk of developing PHN.^4,5,15^ PHN may also be largely refractory to pharmacological treatments.^4,5^ While traditional medicines may also be frequently used for the management of HZ in China, their effectiveness and role in HZ treatment and protection against the development of complications are not well-understood.^13^

The National Medical Products Administration approved the adjuvanted recombinant zoster vaccine (RZV, Shingrix, GSK) in China in May 2019, for use in adults ≥50 YOA to prevent HZ.^16^ This non-live vaccine combines VZV glycoprotein E with the proprietary adjuvant system AS01_B_, which boosts immunogenicity and duration of the immune response.^1^ RZV is to be administered in a two-dose schedule, 2–6 months apart.^16^ Two pivotal phase III trials, ZOE-50 (NCT01165177) and ZOE-70 (NCT01165229), have demonstrated the efficacy of RZV, where RZV reduced the overall incidence of HZ by 97.2% in 7,344 vaccinated participants aged ≥50 years (ZOE-50) and by 91.3% in the pooled analysis (ZOE-70+) of 8,250 vaccinated participants ≥70 YOA from ZOE-50 and ZOE-70.^1^^,17,18^

As RZV is the first vaccine to be approved in China for the prevention of HZ, it is critical to understand the potential public health impact (PHI) of vaccinating the local population with RZV. The objective of this study was to estimate the potential PHI of RZV vaccination, when compared to the status quo of no vaccination, in people aged ≥50 years in Beijing, China, in line with the current RZV label. A Beijing-specific analysis was chosen due to the relevance of available model inputs for HZ disease burden; the city was also of interest as it has been an early leader in vaccination of the elderly population compared with other cities or regions in China, in, for example, flu vaccinations.^19^ Both the private market setting (which is most likely to represent the near-term, post-launch) and the long-term mass vaccination setting were considered by varying vaccination coverage levels, as this was anticipated to increase over time. To further inform vaccination strategy discussions, the analysis of potential impact of RZV vaccination was stratified by several age cohorts in order to identify the age cohort that may benefit most from RZV vaccination.

## Materials and methods

### Model overview

**Figure 1.**
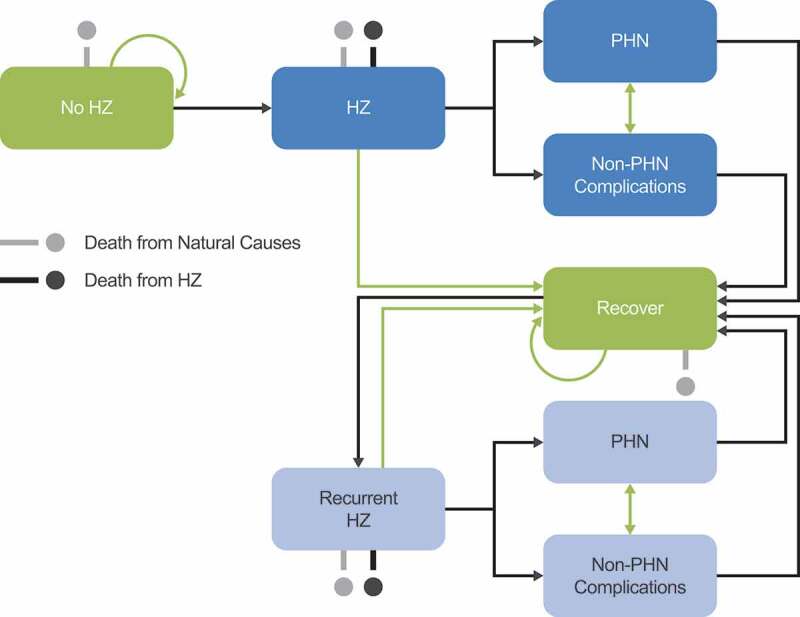
Schematic overview of Markov structure – ZOster ecoNomic Analysis (ZONA) model. HZ: Herpes Zoster; PHN: postherpetic neuralgia. Figure originally published in D. Curran et al. 2017.^20^

This study adapted the ZOster ecoNomic Analysis (ZONA),^20^ a static multi-cohort Markov model with a cycle length of one year, to the Beijing setting. The model followed individuals ≥50 YOA from the year of vaccination (with vaccination being a one-time event for each individual) over their remaining lifetime, stratified into five age cohorts (50–59, 60–64, 65–69, 70–79, and ≥80 YOA). For combined ≥50 YOA results, the model assumed that individuals in each age cohort were vaccinated at 50, 60, 65, 70 or 80 YOA, respectively. The cohorts could transition between different health states, including no HZ, HZ, PHN, HZ-related complications other than PHN, recurrent HZ, HZ-related death, and death due to natural causes (Figure 1).^20^ Annual time steps were used for transition probabilities between health states; PHN and other HZ complications occur during HZ episodes and thus were considered to occur within the same annual time step as the HZ episode. Age-specific probabilities of moving between health states were considered. Further details on the ZONA model may be found in previous publications.^2,20–22^

Two strategies were explored: vaccination with RZV (where individuals could be fully compliant, partially compliant or not vaccinated, based on assumed vaccination coverage and second-dose compliance rates) and the status quo of no vaccination. In the current analysis, results from the 60–64 and 65–69 YOA cohorts were combined to form a 10-year range for consistency in presentation, such that results are presented for four age cohorts: 50–59, 60–69, 70–79, and ≥80 YOA.

### Model input parameters

Wherever possible, Beijing-specific data were used, although where limited, data relating to the whole of China, other cities or regions of China, or neighboring countries were used (Table 1).

**Table 1.** Demographic, epidemiological and vaccine efficacy input values (base case)

Age (years)	50–54	55–59	60–64	65–69	70–74	75–79		80+	Source
Population size of age cohort in 2018	3,310,000		1,234,000	840,000	988,000			586,000	Beijing Statistical Yearbook 2019^23^
All-cause annual mortality probability (%)^†^	0.44	0.56	1.14	1.96	2.94		4.93	80–84: 8.66 85–89: 13.39 90–94: 21.27 95–99: 30.70 100+: 41.95	Global Burden of Disease 2017^24^
HZ incidence (per 1,000 population)	8.03		10.33		11.41			14.00	Jiang et al. 2019^25^
Proportion of HZ cases developing PHN (%)	6.30		10.30		11.40			30.50	Jiang et al. 2019^25^
Proportion of HZ cases developing other HZ-related complications (%)									Jiang et al. 2019^25^
Ocular	0.90		0.70		0.90			0.40	
Neurological (meningitis)	0.30		0.40		0.00			0.20	
Auricular	1.50		2.00		1.70			1.40	
Other	0.60		0.80		1.30			0.60	
HZ-related mortality (% of HZ cases)	0.0000				0.0020		0.0092	80–84: 0.0210 85–89: 0.0467 90–94: 0.1204 95–99: 0.1960 100+: 0.9476	Shiragami et al. 2019^26^
Mean hospitalizations (per HZ case)	0.014		0.026		0.039			0.081	Suo et al. 2019^27^
Mean outpatient visits (per HZ case)^‡^	2.386		2.374		2.361			2.319	
Initial HZ and PHN vaccine efficacy [1-dose]	90.0%				69.5%				van Oorschot et al. 2019;^3^ Curran et al. 2017^20^
Initial HZ and PHN vaccine efficacy [2-dose]	98.4%				97.8%				
Annual RZV waning [1-dose]									van Oorschot et al. 2019;^3^ Curran et al. 2017^20^
Years 1–4 after vaccination	5.4%								
Years 4+ after vaccination	5.1%								
Annual RZV waning [2-dose]									
Years 1–4 after vaccination	1.0%				3.6%				
Years 4+ after vaccination	2.3%								

HZ: herpes zoster; PHN: postherpetic neuralgia; RZV: recombinant zoster vaccine. ^†^The all-cause mortality probability reported for age 100–104 years in the Global Burden of Disease Collaborative Network was used to represent that for age 100+ years in the current analysis. ^‡^The average number of outpatient visits per age group was estimated by subtracting the reported proportion of hospitalizations per HZ case from the reported total 2.4 healthcare visits – which include both outpatient visits and hospitalizations – per HZ case across all age groups.

#### Demographics

The Beijing Statistical Yearbook 2019 provided data on population size.^23^ The target population (adults in Beijing aged ≥50 YOA) comprised approximately 6.96 million individuals. In the absence of local all-cause mortality data for Beijing or China, internationally-consolidated mortality data for China were obtained from the Global Burden of Disease Collaborative Network.^24^

#### Epidemiology

While literature reviews conducted to assess the burden of HZ in China revealed two studies that have assessed HZ disease burden in Beijing, China,^14^,^27^ several data limitations were of concern, including recall bias in Lu et al. 2018, which was a retrospective survey, and selection bias due to database coverage limitations in Suo et al. 2019. Therefore, Jiang et al. 2019,^25^ although conducted in the setting of Yichang city, was chosen as the data source for HZ incidence, as the study was undertaken with an aim to provide baseline data for HZ disease burden in mainland China, specifically in urban settings. At the time of analysis, it was the only published epidemiological study which estimated HZ disease burden by comprehensively employing integrated regional databases, which extensively cover hospitals as well as community health centers.

Estimates for the proportion of HZ cases with complications, including PHN and non-PHN complications (classified as ocular, neurological, auricular, and other), were similarly obtained from Jiang et al. 2019.^25^ International Classification of Diseases (ICD)-10 codes are used as the standard for diagnosis in the Yichang database, which was utilized in Jiang et al. 2019. Therefore, in this study, PHN was likely defined in alignment with the local Chinese expert consensus, which specifies PHN as pain lasting more than one month after cutaneous healing.^28^ The incidence rate of recurrent HZ was assumed to be the same as the incidence of the initial event.^6^,^29^,^30^

Healthcare resource utilization was quantified as mean number of hospitalizations and outpatient visits per HZ case. Suo et al. 2019,^27^ a study conducted in Beijing, was selected as the data source as it provided sufficient data granularity, and as healthcare seeking behavior may differ greatly across cities or regions. As HZ-related mortality data were not readily available for Beijing or China, data from Japan were employed.^26^ The model assumed no additional mortality specifically attributable to PHN or other complications, on top of that from HZ.

#### Vaccine efficacy

An Asian subgroup analysis of global trial data has confirmed a lack of variation in RZV efficacy results by ethnicity in the Asian population.^31^ Therefore, pooled estimates for vaccine efficacy from the ZOE-50 and ZOE-70 global trials were used in this adaptation of the ZONA model to Beijing,^17,18^ as the larger sample sizes lent increased precision. These inputs were previously validated with a panel of experts in epidemiology, modeling and immunology.^20^

Longer-term values for duration of protection for two-dose RZV were extrapolated by applying a linear approximation to annual vaccine efficacy estimates from the four-year duration of the ZOE-50 and ZOE-70 trials, which indicated that waning rates for two-dose RZV were 1.0% (range: 0%–2.6%) annually in the first four years in ZOE-50, and 3.6% (range: 1.4%–6.6%) in ZOE-70+.^20^ Based on these data, it was assumed that for those 50–69 YOA, the vaccine efficacy of RZV would wane at 1.0% per year until year 4 after vaccination, and subsequently at 2.3% (i.e. the average of 1.0% and 3.6%) per year until 69 YOA, and at 3.6% per year for those ≥70 YOA.

As RZV was developed to be administered in a two-dose schedule, no clinical trials have been conducted to assess the efficacy of single-dose RZV. However, it was necessary to consider the efficacy of a single dose to inform outcomes, as compliance with the second dose of RZV is unlikely to be 100% in clinical practice. The only available vaccine efficacy data for one-dose RZV are from a post-hoc analysis conducted using the limited clinical data from subjects who received the first dose of RZV in the ZOE trials; >95% of these subjects later went on to receive the second dose, and the rest only received one dose.^20^ Given the mean follow-up period for subjects who received only the first dose was less than three months, long-term vaccine efficacy data for one dose of RZV are unavailable. In the absence of these data, it was assumed that RZV would wane at the same rate as Zoster Vaccine Live (ZVL): 5.4% for the initial four years, and 5.1% thereafter.^20^

Overall vaccine efficacy against PHN was assumed to be equal to vaccine efficacy against HZ (i.e. no top-up efficacy against PHN), as high vaccine efficacy led to very few breakthrough cases in the ZOE trials, with only four cases of PHN observed altogether (zero in the ZOE-50 and four in the ZOE-70 vaccination groups).^18^

Vaccine efficacy over time was calculated as a function of age-specific response to vaccine, waning rate of efficacy, time step (age-specific function), the number of doses, the dosing schedule and uptake of the first and second vaccine dose. The two doses of RZV were assumed to be separated by a two-month interval.

Across all age cohorts, the base-case vaccination coverage was assumed to be 5% in the private market setting (near-term post-launch), while a vaccination coverage of 50% was assumed for the mass vaccination setting (long-term), based on publicly-funded influenza vaccination uptake in Beijing among older adults.^19^

It was expected that second-dose compliance in Beijing would be higher than has been observed elsewhere (e.g. about 65% in Canada)^22^ because of the local population’s acceptability toward HZ prevention, and the absence of other preventive measures such as ZVL. Nevertheless, second-dose compliance was also expected to be lower than that observed in the ZOE-50 and ZOE-70 clinical trials (about 95%).^17,18^ Second-dose compliance of RZV was thus assumed to be 80% (the mid-point of 65% and 95%) in the base case for both market settings.

### Outcomes

Key health outcomes of interest estimated by the model were the number of HZ cases, number of PHN cases and other HZ-related complications, healthcare resource utilization (number of outpatient visits and hospitalizations), and HZ-related deaths. The numbers accumulated over the entire time horizon were calculated for each strategy (RZV vaccination and the status quo of no vaccination) and compared. The potential impact of RZV vaccination was then presented as stratified by four age cohorts (50–59, 60–69, 70–79, and ≥80 YOA). Numbers of individuals needed to vaccinate (NNV) were also calculated for avoiding one HZ case and for avoiding one PHN case, respectively. NNVs were obtained by applying the following calculation:^3^

$$NNV=1/\left(\left(\frac{Control\;cases}{Vaccinated\;persons}\right)-\left(\frac{Vaccinated\;cases}{Vaccinated\;persons}\right)\right)$$

### Sensitivity analyses

Sensitivity analyses were conducted on the mass vaccination setting only, for the primary outcome of interest (HZ cases avoided). A deterministic one-way sensitivity analysis (DSA) was conducted to assess the robustness of the results to uncertainties around the input parameters by varying each of the model’s key input variables one at a time, over either reported ranges (e.g. 90% confidence intervals) or ±20% of the base-case estimates (**Supplementary Table 1**).

A probabilistic sensitivity analysis (PSA) was also undertaken to account for the full uncertainty in model inputs and explore its impact on the primary outcome. Monte Carlo simulations (5,000 iterations) were run, where input values were simultaneously sampled from probability distributions (**Supplementary Table 1**). All parameters were sampled across beta-distributions, and age-specific incidence parameters were assumed to have a correlation of 0.5.

### Scenario analyses

Two scenario analyses were performed for the primary outcome of interest (HZ cases avoided): in the first scenario analysis, inputs for vaccination coverage (5%, 10%, 30%, 40%, 50%) and second-dose compliance (60%, 70%, 80%, 90%, 100%) were varied concomitantly. In the second scenario analysis, conducted on the mass vaccination setting only, parameters simultaneously varied were second-dose compliance for RZV, efficacy and waning of one-dose and two-dose RZV.

## Results

The potential PHI of RZV vaccination was explored under two market settings: private market and mass vaccination. As the only difference between the private market and mass vaccination settings was the assumed vaccination coverage level (5% vs. 50%), the results for the former market setting can be obtained by dividing those for the latter setting by 10, except for NNV results, which are not affected by the vaccination coverage level. This section will focus on presenting the results under the mass vaccination setting only; values for the private market setting may be found in **Supplementary Table 2**.

### Base-case analysis

In the base case and under the mass vaccination setting, RZV vaccination in Beijing reduced the number of HZ and PHN cases by 435,681 and 51,558 respectively in the overall ≥50 YOA cohort over the entire time horizon, compared with no vaccination (Tables 2 and 3). 15,703 cases of other HZ-related complications would also be avoided in the overall ≥50 YOA cohort with mass RZV vaccination, compared with no vaccination (Tables 2 and 3). RZV vaccination had the greatest impact on the 50–59 YOA cohort, which was responsible for 54.7% of the total HZ case reduction, 41.5% of the total PHN case reduction, and 54.6% of the total other HZ-related complications avoided in the overall ≥50 YOA cohort (Table 3).

**Table 2.** Health outcomes and healthcare resource utilization in individuals ≥50 YOA (base case)

	No vaccination		Private market setting (RZV vaccination coverage: 5%)			Mass vaccination setting (RZV vaccination coverage: 50%)		
	Cases		Cases	Cases avoided		Cases		Cases avoided
HZ	1,654,291		1,610,723	43,568		1,218,610		435,681
PHN	249,525		244,369	5,156		197,967		51,558
HZ-related complications other than PHN	57,572		56,002	1,570		41,870		15,703
Ocular	11,893		11,556	337		8,523		3,370
Neurological (meningitis)	3,450		3,345	105		2,403		1,048
Auricular	27,744		26,993	751		20,237		7,507
Other	14,485		14,107	378		10,706		3,779
HZ-related deaths	162		160	2		143		19
Healthcare resource utilization								
Hospitalizations	69,470	68,045			1,425	55,223	14,247	
Outpatient visits	3,900,829	3,797,690			103,139	2,869,441	1,031,387	

HZ: herpes zoster; PHN: postherpetic neuralgia; RZV: recombinant zoster vaccine; YOA: years of age. The total cohort vaccinated was 347,900 in the private market setting and 3,479,000 in the mass vaccination setting analysis. Small discrepancies between subgroups and total cases may be present due to rounding.

**Table 3.** Cases avoided with RZV vaccination vs. no vaccination by age cohort, in the mass vaccination setting (base case)

	Age cohort (years)				
N (%)	50–59	60–69	70–79	80+	Total (≥50 years)
HZ cases avoided	238,301 (54.7%)	132,179 (30.3%)	45,100 (10.4%)	20,101 (4.6%)	435,681 (100.0%)
PHN cases avoided	21,397 (41.5%)	16,818 (32.6%)	7,213 (14.0%)	6,131 (11.9%)	51,558 (100.0%)
HZ-related complications other than PHN avoided	8,578 (54.6%)	4,984 (31.7%)	1,618 (10.3%)	523 (3.3%)	15,703 (100.0%)
Ocular	1,952 (57.9%)	985 (29.2%)	352 (10.4%)	80 (2.4%)	3,370 (100.0%)
Neurological (meningitis)	683 (65.2%)	303 (28.9%)	22 (2.1%)	40 (3.8%)	1,048 (100.0%)
Auricular	4,076 (54.3%)	2,415 (32.2%)	734 (9.8%)	281 (3.7%)	7,507 (100.0%)
Other	1,868 (49.4%)	1,280 (33.9%)	510 (13.5%)	121 (3.2%)	3,779 (100.0%)
HZ-related deaths avoided^†^	3 (13.3%)	6 (30.6%)	5 (24.6%)	6 (31.5%)	19 (100.0%)
Healthcare resource utilization avoided					
Hospitalizations	5,597 (39.3%)	4,807 (33.7%)	2,214 (15.5%)	1,628 (11.4%)	14,247 (100.0%)
Outpatient visits	566,325 (54.9%)	312,422 (30.3%)	106,026 (10.3%)	46,615 (4.5%)	1,031,387 (100.0%)

HZ: herpes zoster; PHN: postherpetic neuralgia; RZV: recombinant zoster vaccine; YOA: years of age.

The total cohort vaccinated was 3,479,000 in the mass vaccination setting. Small discrepancies between subgroups and total cases may be present due to rounding.

^†^A greater number and proportion of HZ-related deaths would be avoided in the older cohorts compared to the 50–59 YOA cohort, as (i) the estimated HZ-related death probability remains as 0% in the current model until the cohort reaches 70 YOA and older, (ii) RZV efficacy may wane over time, and (iii) not all vaccinated individuals in the 50**–**69 YOA cohort will reach 70**–**79 or 80+ YOA

In terms of healthcare resource utilization, in the mass vaccination setting, 14,247 hospitalizations and 1,031,387 outpatient visits could be avoided by vaccinating individuals ≥50 YOA with RZV (Table 2). Younger age cohorts, in particular the 50–59 YOA cohort, had greater contributions to the overall reduction in HZ-associated healthcare resource utilization (Table 3). The current analysis estimated few HZ-related deaths overall; compared with no vaccination, RZV vaccination would prevent 19 HZ-related deaths (Table 2).

**Figure 2.**
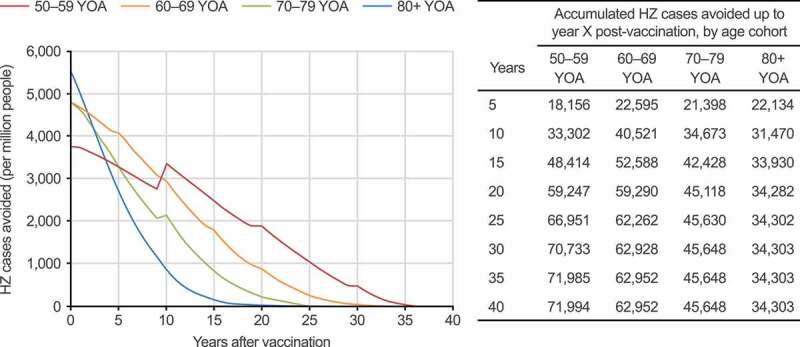
HZ cases avoided with RZV vaccination per one million people over the respective age cohorts’ lifetime, compared with no vaccination, in the mass vaccination setting (base case). HZ: herpes zoster; RZV: recombinant zoster vaccine; YOA: years of age. Step increases over time (particularly pronounced in the 50–59 YOA cohort) are due to incidence being included as an age-specific step function

An analysis of HZ cases avoided per one million people was conducted, which removed the impact of varying population sizes across age cohorts (Figure 2). Over the lifetime of these cohorts, the greatest benefit associated with HZ avoidance following RZV vaccination was expected to occur in the first few years and decrease over time, with short-term effects being more pronounced for the older cohorts. In the long term, the largest impact on cumulative HZ cases avoided was still seen in the 50–59 YOA cohort (in the mass vaccination setting: 71,994 HZ cases prevented per one million people over the cohort’s lifetime, vs. 34,303 in the 80+ YOA cohort).

**Figure 3.**
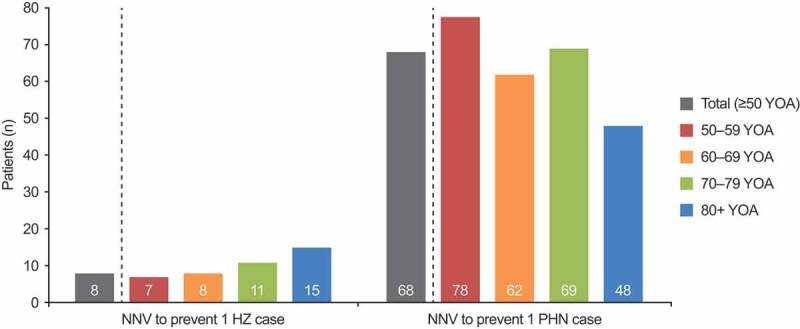
NNV to avoid one case of HZ and one case of PHN by age cohort (base case). HZ: herpes zoster; NNV: number needed to vaccinate; PHN: postherpetic neuralgia; YOA: years of age. NNV results are not affected by the vaccination coverage level, and thus do not differ between the private market and mass vaccination settings

The NNV to prevent one case of HZ and PHN was estimated to be 8 and 68, respectively (Figure 3). The NNV to prevent one HZ case increased with age, from 7 in the 50–59 YOA cohort to 15 in the 80+ YOA cohort. For PHN cases, the NNV decreased from 78 in the 50–59 YOA cohort to 48 in the 80+ YOA cohort; however, no clear association was seen between age and NNV when considering the 70–79 YOA cohort.

### Sensitivity analyses

**Figure 4.**
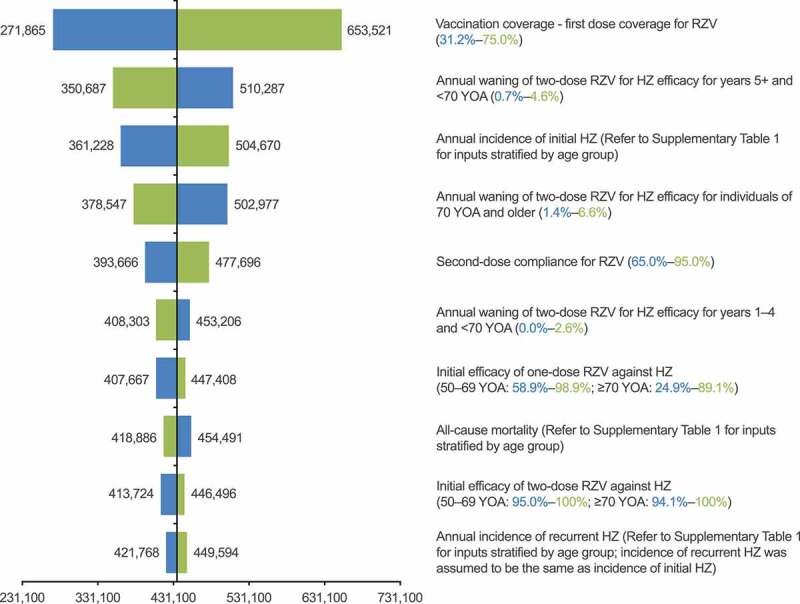
Deterministic one-way sensitivity analysis: HZ cases avoided with RZV vaccination, compared with no vaccination. HZ: herpes zoster; RZV: recombinant zoster vaccine; YOA: years of age. Lower bound estimates are in blue and upper bound estimates are in green, with lower and upper ranges for inputs in brackets; refer to Supplementary Table 1 for details of inputs which varied by age group

**Figure 5.**
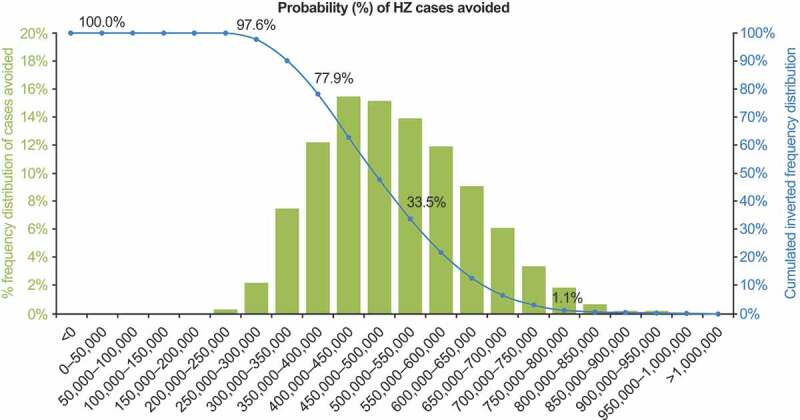
Probabilistic sensitivity analysis: HZ cases avoided with RZV vaccination, compared with no vaccination. HZ: herpes zoster. The blue line shows the percentage of simulations averting at least the number of HZ cases shown on the x-axis

The DSA results are summarized in the tornado diagram presented in Figure 4. The parameter with the highest impact on the number of HZ cases avoided by RZV vaccination was vaccination coverage; nevertheless, even the lowest coverage rate explored in the DSA (31.2%) would still lead to a reduction of over 270,000 HZ cases compared with the status quo of no vaccination. In the PSA, as summarized in the histogram, 77.9% of the 5,000 simulations predicted that RZV vaccination would prevent at least 350,000 HZ cases compared with no vaccination among individuals aged ≥50 YOA (Figure 5).

### Scenario analyses

**Figure 6.**
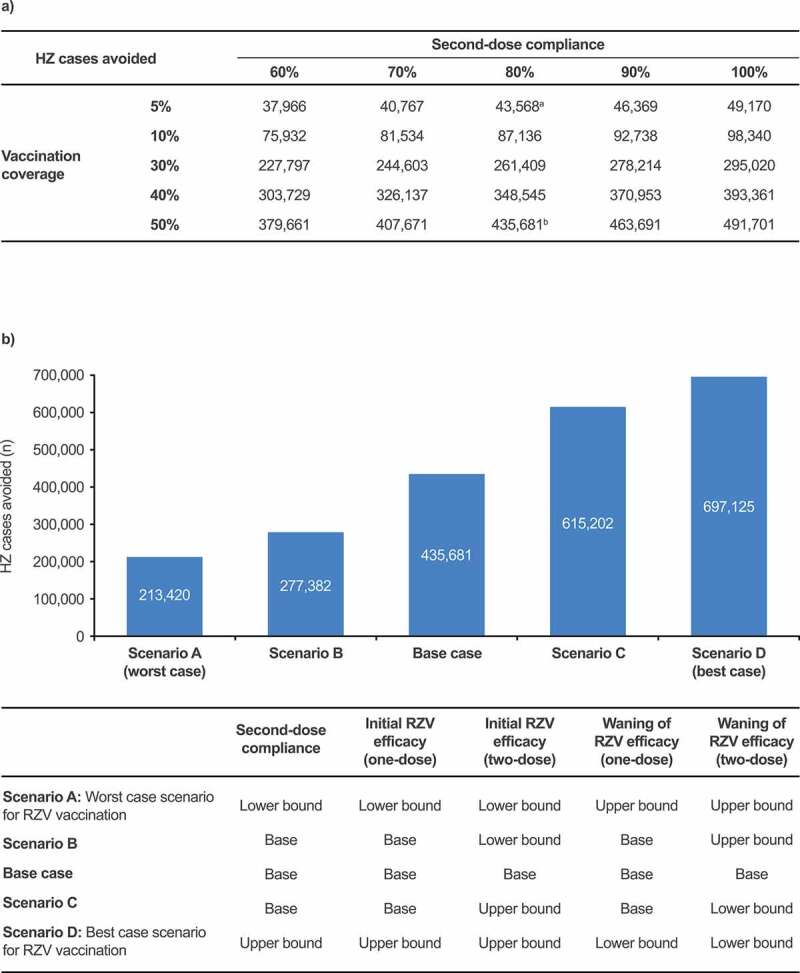
Scenario analysis assumptions and results comparing RZV vaccination with no vaccination, targeting individuals 50 YOA and older, estimating the number of HZ cases avoided (a) with increasing vaccination coverage and second-dose compliance; (b) with varying second-dose compliance, RZV efficacy, and its waning. ^a^Base-case outcome for the private market setting analysis. ^b^Base-case outcome for the mass vaccination setting analysis. Values for lower and upper bounds can be found in Supplementary Table 1. HZ: herpes zoster; PHN: postherpetic neuralgia; RZV: recombinant zoster vaccine; YOA: years of age

The first scenario analysis demonstrated that a higher level of vaccination coverage and the two-dose RZV vaccination, compared to one-dose RZV vaccination, could provide increased public health benefits (Figure 6a). The second scenario analysis, which explored permutations of the estimates for second-dose compliance, RZV efficacy and its waning, suggested that in the most optimistic situation, approximately 697,125 HZ cases could be avoided using RZV vaccination compared with no vaccination, yielding an additional 261,444 HZ cases to be avoided compared with the base case. In the most pessimistic situation, 213,420 HZ cases could still be avoided (Figure 6b).

## Discussion

Adapting an existing validated Markov model,^20^ this analysis demonstrated the value of RZV vaccination in reducing the burden of HZ, HZ-related complications, and healthcare resource utilization among adults aged ≥50 YOA in Beijing, compared with the status quo of no vaccination. Vaccination in the 50–59 YOA cohort contributed to the largest numbers of cases avoided in the overall ≥50 YOA cohort.

In the base case and mass vaccination setting, RZV vaccination reduced the number of HZ and PHN cases by 435,681 and 51,558, respectively, in the overall ≥50 YOA cohort over the entire time horizon, compared with no vaccination. HZ-related complications other than PHN were also expected to substantially decline as a result of RZV vaccination. The significant contribution from the 50–59 YOA cohort to the overall reduction in HZ cases and its complications is likely due to the 50–59 YOA cohort’s large population size, the relatively low all-cause mortality rate, and the projected long-term protection offered by RZV vaccination.^1^ In the long term, even when the larger population size of the younger cohort was factored out, the greatest reduction of HZ was expected for the 50–59 YOA cohort compared with older cohorts. Vaccinating at younger ages, as young as 50 YOA, may thus have a greater effect on avoidance of HZ, PHN and other HZ-related complications than targeting older cohorts.

In terms of healthcare resource utilization, 14,247 hospitalizations and 1,031,387 outpatient visits could be avoided by the mass vaccination of individuals 50 YOA and older with RZV. Although fewer hospitalizations are expected to be avoided compared with outpatient visits, the former could be understood as a proxy for severe HZ episodes,^32^ which may incur greater direct medical costs per individual case. Furthermore, with the substantial contributions to avoided HZ cases and associated healthcare resource use expected from the 50–59 YOA cohort, early RZV vaccination may result in additional cost-related benefits by reducing productivity loss, as a sizable proportion of the 50–59 YOA cohort are likely to still be part of the workforce.^33^ The impact of RZV vaccination on direct and indirect costs should be further investigated through a robust local disease burden study and additional modeling analysis.

The NNV to avoid a case of HZ ranged from 7 to 15 between age cohorts, and increased with age. However, no clear association was seen between age and the NNV for preventing one PHN case across all age cohorts, specifically in considering the 70–79 YOA cohort. The NNV to prevent one PHN case was lowest for the ≥80 YOA cohort, due to the proportion of HZ cases with PHN being estimated at 30.5% in this oldest age cohort, which is substantially higher than the proportions assumed in the younger age groups in the model.

Sensitivity analyses showed that results are considered robust under both DSA and PSA. The scenario analyses highlighted the importance of increasing vaccination coverage and completion of the RZV vaccination schedule in increasing the PHI of RZV vaccination.

The study results may aid local clinicians and policy makers in their value assessment of RZV vaccination, for example in recommending vaccination with RZV as early as 50 YOA as a long-term HZ prevention strategy in local consensus statements or guidelines. However, it should be acknowledged that factors considered in the current analysis such as demographics may change over time, impacting recommendations regarding vaccination strategy; for instance, in an aging population with an increasing number of adults ≥60 YOA,^13^ RZV vaccination for the older cohort may be prioritized with an aim to achieve rapid HZ avoidance. Furthermore, factors other than those that have been accounted for in the current PHI analysis may be considered for value assessment and decision-making. This may include the cost burden, although data on the cost burden associated with HZ in China, including direct and indirect costs, remain limited at both individual and population levels.^13^ Patients’ quality of life may be another critical consideration in vaccination strategy; for instance, the quality of life of older patients may be more negatively impacted by HZ compared with younger cohorts.^4^ Future research to assess and quantify various aspects of the local HZ disease burden would help to guide experts in making better-informed assessments and recommendations for RZV vaccination in China. These data would also enable future modeling analyses such as a cost-effectiveness analysis, which was found to be difficult to undertake at the time of the current study due to a lack of relevant local data.

### Strengths and limitations

This study is the first PHI analysis for RZV vaccination in mainland China. The robustness of the ZONA model is supported by several previous publications reporting similar analyses in other countries.^2,20,21,34^ For this study, the most recent data appropriate to the Beijing context (urban settings in mainland China) were used as model inputs, and the model was adapted to present potential scenarios relevant to both near-term post-launch (private market) and long-term (mass vaccination) market settings. Vaccine efficacy inputs and assumptions used were in line with those of Curran et al. 2016, whose methods were validated during an advisory board meeting with international experts.^20^

Limitations of this study include the lack of Beijing-specific data for some inputs. For instance, all-cause mortality data for China was used, likely resulting in a conservative analysis outcome (i.e. more HZ cases could be avoided than is shown), as the average life expectancy for the population in Beijing is generally higher than that for China.^23,24^^,35^ More people who had been immunized with RZV could thus have benefitted from vaccination over a longer time period.

Other limitations include the uncertainty regarding vaccination coverage, second-dose compliance, and the RZV efficacy waning rate, as the lack of available real-world data required assumptions to be made. To mitigate this, vaccine efficacy waning rates and their upper and lower bounds were validated by a group of international experts,^20^ and the parameters were extensively evaluated in sensitivity and scenario analyses. Even in the most pessimistic scenario explored, a substantial number of HZ cases are expected to be avoided. Finally, due to evidence limitations, the same probability for HZ-related complications was assumed regardless of vaccination status, disregarding a potential impact of vaccination on HZ severity. This conservative approach was taken despite some evidence indicating that the duration and severity of pain in vaccinated individuals may be lower compared with unvaccinated individuals.^36^

This analysis demonstrates the potential PHI of RZV vaccination, when compared with the status quo of no vaccination, among individuals aged 50 years and older in Beijing, China. Vaccination with RZV, especially in the mass vaccination setting, can be expected to substantially reduce HZ cases, HZ-related complications, and healthcare resource utilization compared with no vaccination. The 50–59 YOA cohort was observed to provide the greatest contribution to the reduction in cases and resource use. Vaccination with RZV as early as 50 YOA may be a prudent long-term HZ prevention strategy, although other factors such as cost burden and impact on quality of life should also be given due consideration.

Although the current analysis was undertaken specifically for Beijing, the results and insights obtained may also be pertinent to other cities or regions of China with comparable population sizes and relevant market contexts (e.g. private or mass vaccination), where unmet needs regarding the prevention of HZ exist.

## Supplementary Material

Supplemental Material

## Figures and Tables

**Figure 7.**
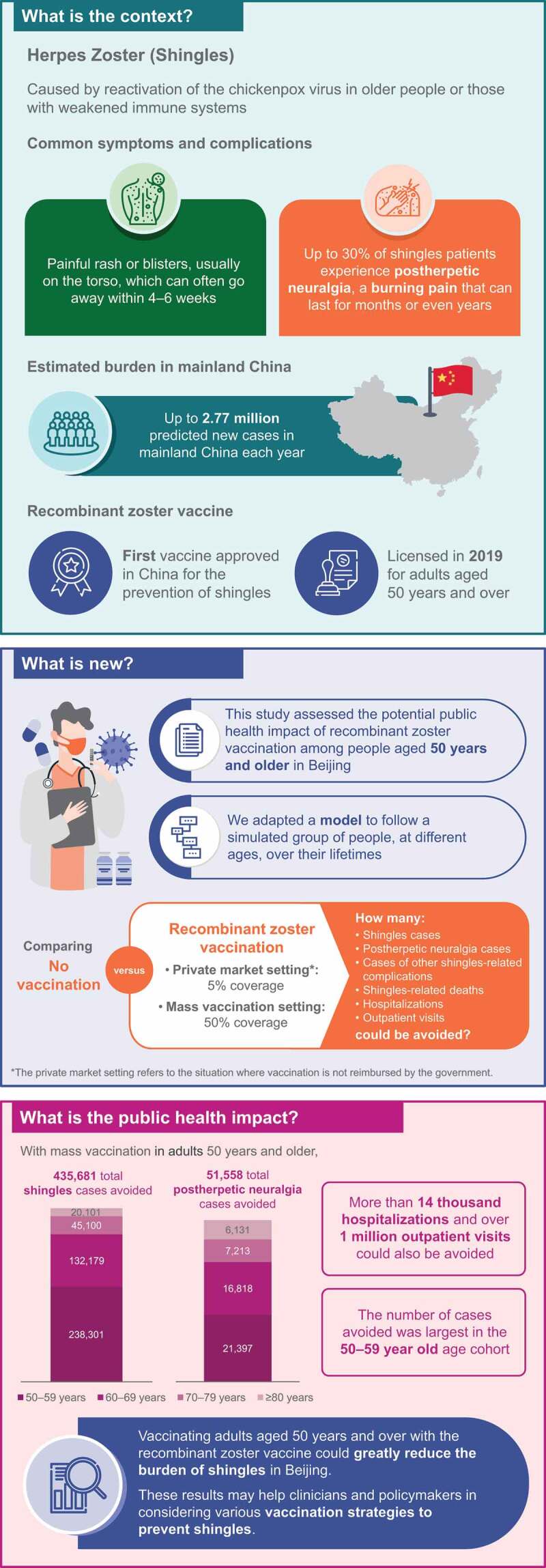
Plain language summary

## Note

1 Participant numbers are taken from the modified vaccinated cohorts, which excluded participants who did not receive the second dose of vaccine or who received a confirmed diagnosis of HZ within 1 month after the second dose.

## Trademark

Shingrix is a trademark of the GSK group of companies.
